# Silicon transport and its “homeostasis” in rice

**DOI:** 10.1017/qpb.2024.19

**Published:** 2025-01-09

**Authors:** Sheng Huang, Jian Feng Ma

**Affiliations:** 1 Institute of Plant Science and Resources, Okayama University, Kurashiki, Japan

**Keywords:** deposition, homeostasis, intervascular transfer, modelling, rice, silicon, transporter, uptake

## Abstract

Silicon (Si), the most abundant mineral element in soil, functions as a beneficial element for plant growth. Higher Si accumulation in the shoots is required for high and stable production of rice, a typical Si-accumulating plant species. During the last two decades, great progresses has been made in the identification of Si transporters involved in uptake, xylem loading and unloading as well as preferential distribution and deposition of Si in rice. In addition to these transporters, simulation by mathematical models revealed several other key factors required for efficient uptake and distribution of Si. The expression of *Lsi1*, *Lsi2* and *Lsi3* genes is down-regulated by Si deposition in the shoots rather than in the roots, but the exact mechanisms underlying this down-regulation are still unknown. In this short review, we focus on Si transporters identified in rice and discuss how rice optimizes Si accumulation (“homeostasis”) through regulating Si transporters in response to the fluctuations of this element in the soil solution.

## Graphical abstract

**Figure:**
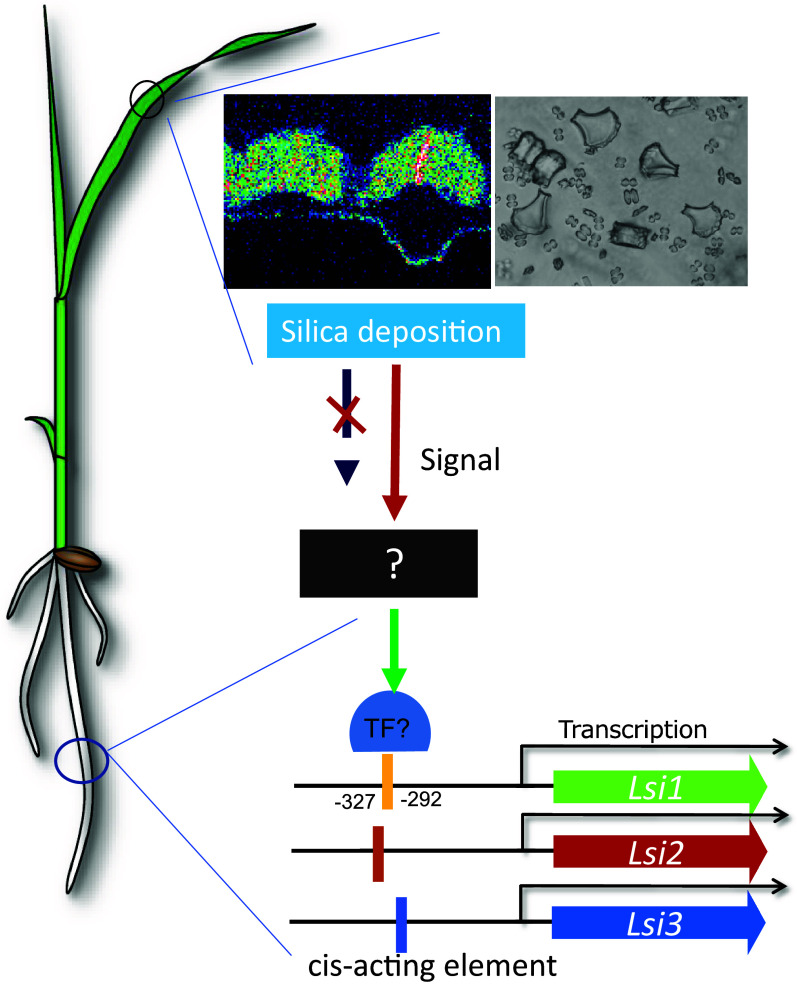

## 1. Introduction

Silicon (Si) is the most abundant mineral element in the earth’s crust and all plants rooted in soil contain substantial amounts in their tissues (Epstein, 1999). However, the Si accumulation in the above-ground parts differs greatly among plant species, ranging from 0.1% to 10% of the dry weight (Ma & Takahashi, 2002; Hodson et al., 2005). For example, rice, a typical Si-accumulating plant, is able to accumulate Si in the shoots up to 10%, whereas tomato plant only can accumulate 0.2% even when grown under the same conditions (Ma & Takahashi, 2002). These large differences in Si accumulation have been associated with Si transporters as detailed below.

Silicon has not been recognized as an essential element because no evidence on its involvement in the metabolism has been found, which is one of the criteria for the essentiality of elements to plants (Arnon & Stout, 1939). However, multiple beneficial effects have been reported for a number of plant species differing in Si accumulation, which are characterized by mitigating various biotic and abiotic stresses (Debona et al., 2017; Debona et al., 2023; Ma & Takahashi, 2002; Ma, 2004; Coskun et al., 2019). For example, Si increases the resistance to herbivores and microbial pathogens (Coskun et al., 2019). Silicon is also able to enhance the tolerance to metal toxicity, nutrient imbalance and lodging (Ma, 2004). Silicon is especially important for high and stable production of rice, and insufficient accumulation in the shoots and husk significantly decreases rice yield (Tamai & Ma, 2008). Therefore, Si has been recognized as an ‘agronomically essential element’ for rice (Ma & Takahashi, 2002). Several mechanisms for the beneficial effects of Si have been proposed (Debona et al., 2017; Debona et al., 2023; Coskun et al., 2019), but the major one is the formation of a physical barrier due to Si deposition at different tissues, which protects the plants from biotic and abiotic stresses (Ma & Yamaji, 2006). Therefore, to benefit from Si, it must be transported from the soil to different organs and tissues. During the last two decades, transporters involved in different transport steps have been identified, especially in rice. In this short review, we focus on these transporters in rice, an important staple food and a model plant of cereal crops. We also discuss how rice maintains Si “homeostasis” in rice.

**Figure 1.**
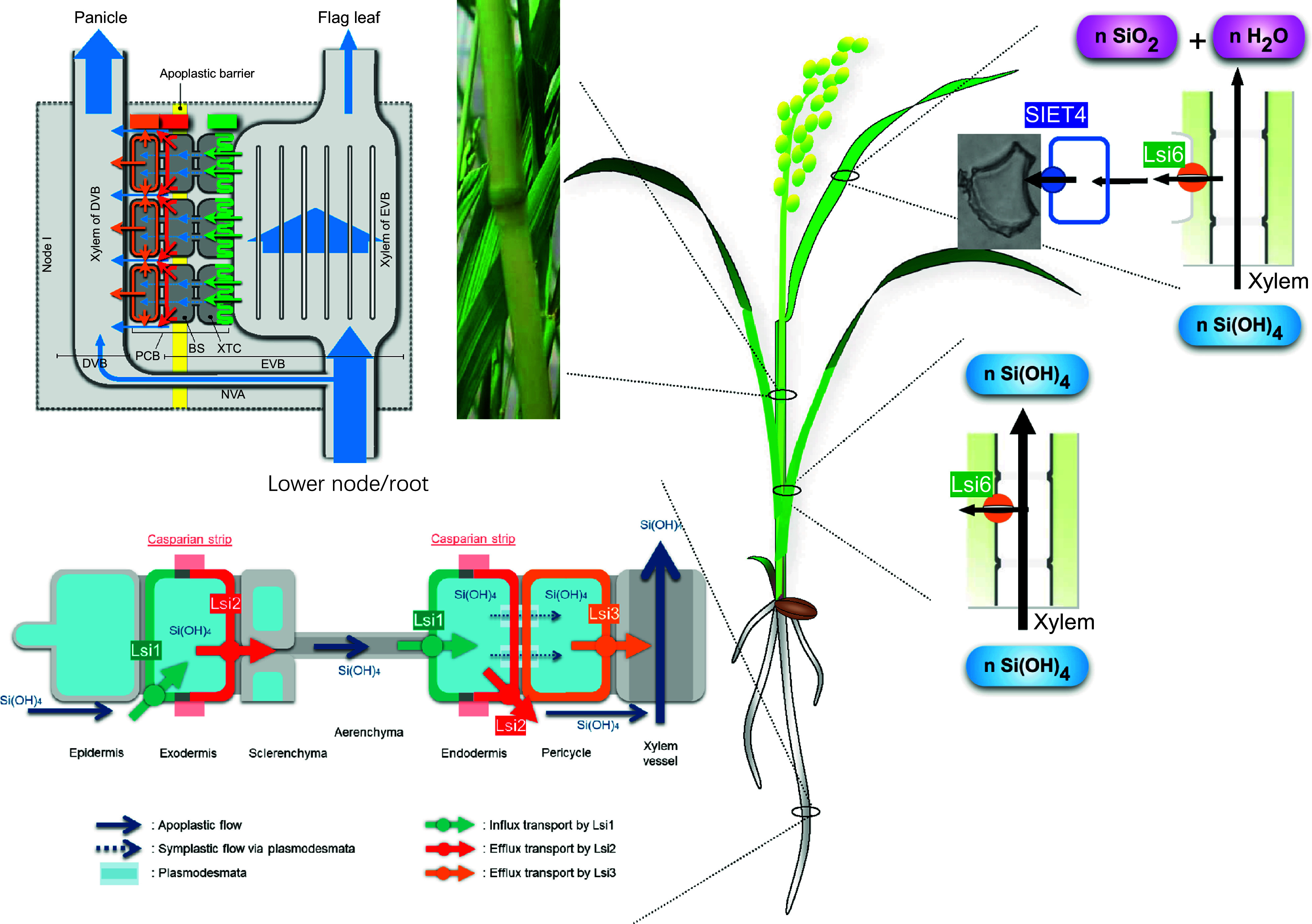
Overview of silicon (Si) transporters involved in uptake, xylem loading and unloading, preferential distribution, and its deposition in rice. Silicon as monosilicic acid is taken by Lsi1 and Lsi2, polarly localized at the root exodermis and endodermis. Xylem loading of Si as silicic acid is mediated by Lsi3 localized at the root pericycle, while xylem unloading is by Lsi6 localized at the xylem parenchyma cells. The deposition of Si to special cells and tissues is mediated by SIET4. Preferential distribution of Si to the grain is facilitated by three transporters; Lsi6, Lsi2 and Lsi3 located at the different cell layers in node I.

## 2. Transport system of Si in rice

### 2.1. Transporters involved in Si uptake

Silicon in the soil solution is present in the form of silicic acid {Si(OH)_4_} at a pH under 9, a non-charged molecule. This form is taken up by the roots through two different transporters; Lsi1 and Lsi2. Lsi1 belongs to the Nod26-like major intrinsic protein (NIP) subfamily of aquaporin-like proteins and functions as an influx transporter of Si (Ma et al., 2006), while Lsi2 belongs to a putative anion transporter family without any similarity to Lsi1 (Ma et al., 2007) and function as an efflux transporter of Si. Both Lsi1 and Lsi2 are highly expressed in the root mature region, but not in the root tip, therefore, the root mature region rather than the root tip is the site of Si uptake (Yamaji & Ma, 2007). Furthermore, Lsi1 and Lsi2 are highly expressed in the lateral roots, but not in root hairs (Ma et al., 2006; Yamaji & Ma, 2007), indicating that root hairs do not play a role in Si uptake (Ma et al., 2001). Both Lsi1 and Lsi2 are localized at the exodermis and endodermis in the mature root regions but show different polar localization. Lsi1 is localized at the distal side, while Lsi2 is localized at the proximal side (Figure 1) (Ma et al., 2006; Yamaji & Ma, 2007). Given the distinct root anatomy of rice, silicic acid is first imported into the symplast by Lsi1 at the distal side of the exodermal cells and then exported by Lsi2 at the proximal side to the apoplastic connections (Ma & Yamaji, 2015). Silicic acid is further imported into the symplast of the endodermis by Lsi1 localized at the distal side of the endodermis and is exported to the stele by Lsi2 localized at the proximal side of the endodermis. Therefore, Lsi1-Lsi2 forms an efficient uptake system in rice roots (Figure 1) (Ma & Yamaji, 2015). Recently, the polar localization of Lsi1 was found to be important for the efficient uptake (Konishi et al., 2023) and several critical amino acid residues for the polar localization have been identified. Since the identification of Lsi1 and Lsi2 in rice, similar transporters involved in Si uptake have been identified in other plant species differing in Si accumulation. Analysis of these transporters showed that the large difference in Si accumulation is attributed to the differential expression level, localization, and polarity of these transporters (Mitani-Ueno & Ma, 2021).

### 2.2. Transporters of Si for xylem loading and unloading

Silicon as silicic acid is loaded into the root xylem by Lsi3 (Huang et al., 2022), while unloaded from the xylem by Lsi6 (Figure 1) (Yamaji et al., 2008). Similar to Lsi2, Lsi3 also functions as a Si efflux transporter. However, different from Lsi2, Lsi3 is localized at the root pericycle cells without polarity. Knockout of *Lsi3* resulted in a 20–30% reduction in xylem Si concentration (Huang et al., 2022). On the other hand, Lsi6 is a homolog of Lsi1 and is also permeable to monosilicic acid (Yamaji et al., 2008). Lsi6 is polarly localized at the adaxial side of the xylem parenchyma cells in the leaf sheaths and leaf blades. Knockout of *Lsi6* resulted in increased Si in the guttation fluid and abnormal Si deposition (Yamaji et al., 2008).

### 2.3. Transporters responsible for Si distribution

High accumulation of Si in the husk is required for high rice production. The preferential distribution of Si to the grain is mediated by three different Si transporters; Lsi6, Lsi2, and Lsi3, which are highly expressed in the nodes, especially node I (Figure 1) (Yamaji & Ma, 2009; Yamaji 2015). Lsi6 is localized at the xylem transfer cells of the enlarged vascular bundles, which is responsible for the unloading of Si from the xylem, while Lsi2 and Lsi3 are localized at the bundle sheath and parenchyma bridge cells, respectively, which are responsible for further transfer of Si to the diffuse vascular bundles. Therefore, Lsi6-Lsi2-Lsi3 localized at different cell layers of node I forms an efficient system of Si distribution (Figure 1) (Yamaji & Ma, 2009; Yamaji et al., 2015). Knockout of *Lsi6*, *Lsi2*, or *Lsi3* results in the decreased distribution of Si to the panicles but increased Si to the flag leaf (Yamaji & Ma, 2009; Yamaji et al., 2015).

**Figure 2.**
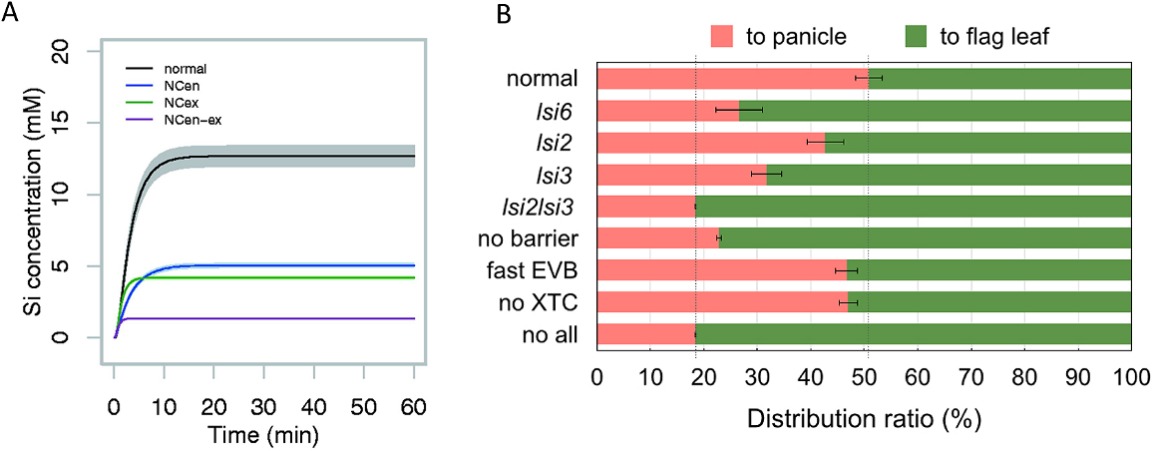
Simulation of silicon (Si) transport system in rice. (A) Role of Casparian strips in Si uptake in rice roots. A time-dependent Si concentration in the xylem sap was simulated for the normal rice and rice with various modified Casparian strip patterns: no Casparian strip in the endodermis (NCen), in the exodermis (NCex) or in both tissues (NCen–ex). Data were obtained from Sakurai et al. (2015). (B) Estimated Si distribution ratio to the panicle and flag leaf by a mathematic model. Normal, normal setting as wild-type rice; *lsi6*, lack of Lsi6; *lsi2*, lack of Lsi2; *lsi3*, lack of Lsi3; *lsi2lsi3*, lack of both Lsi2 and Lsi3; no barrier, no apoplastic barrier at the bundle sheath of EVB; fast EVB, 10× faster velocity of the xylem in EVB; no XTC, permeability parameter of Lsi6 replaced by that of Lsi1 in root; no all, combined defect of all above factors. Data were obtained from Yamaji et al. (2015).

### 2.4. Transporter required for Si deposition

Silicon is finally deposited at different plant tissues as amorphous silica (Ma & Takahashi, 2002). In rice, with transpiration, the concentration of silicic acid in the above-ground tissues increases and then polymerized to silica (SiO_2_), which is deposited beneath the cuticle of leaves and inside particular cells of leaf epidermis, forming silica cells and silica bodies or silica bulliform cells (motor cells) (Ma & Takahashi, 2002). Recently, SIET4, a transporter belonging to the same family of Lsi2 and Lsi3, was found to be involved in Si deposition (Mitani-Ueno et al., 2023). SIET4 is polarly localized at the distal side of epidermal cells and cells surrounding the bulliform cells of the leaf blade, where Si is deposited. Knockout of *SIET4* led to the death of rice grown in soil or nutrient solution containing Si but did not affect plant growth in the absence of Si. Its knockout induced abnormal Si deposition in the mesophyll cells, resulting in the expression of hundreds of genes involved in various biotic/abiotic stress responses although Si uptake and accumulation were not affected (Mitani-Ueno et al., 2023). Therefore, SIET4 is responsible for the proper deposition of Si by exporting this element from leaf cells to the leaf surface, which is required for the adequate growth of rice plants.

## 3. Quantitative analysis of Si transport by mathematical modelling

Quantitative simulation of Si uptake has been made by using a mathematical model based on *in vivo* experimental data (Sakurai et al., 2015). According to this model, in addition to Lsi1 and Lsi2, Casparian strips (CS) at the exodermis and endodermis are also required for high Si uptake in rice (Figure 2A; Sakurai et al., 2015). Furthermore, the localization of Lsi1 and Lsi2 at the exodermis and endodermis and their different polarity are the most efficient patterns for efficient Si uptake. These findings were supported by subsequent experimental evidence. For example, the lack of CS at the endodermis significantly decreased Si uptake due to less abundance of Lsi1 protein (Wang et al., 2019). Plants with polar localization of Lsi1 showed higher Si uptake than those with non-polar localization (Konishi et al., 2023). Furthermore, a 3D root model simulation resulted in the identification of a missing transporter, Lsi3 involved in the xylem loading of Si (Huang et al., 2022). The simulation showed that Lsi3 contributed to a maximum of 30% at around 0.3 mM Si in the external solution (Huang et al., 2022). These findings indicated that rice plants have developed a remarkably effective system of Si uptake and xylem loading during their evolution, which is necessary for the high accumulation of this element in the shoots for adequate growth.

**Figure 3.**
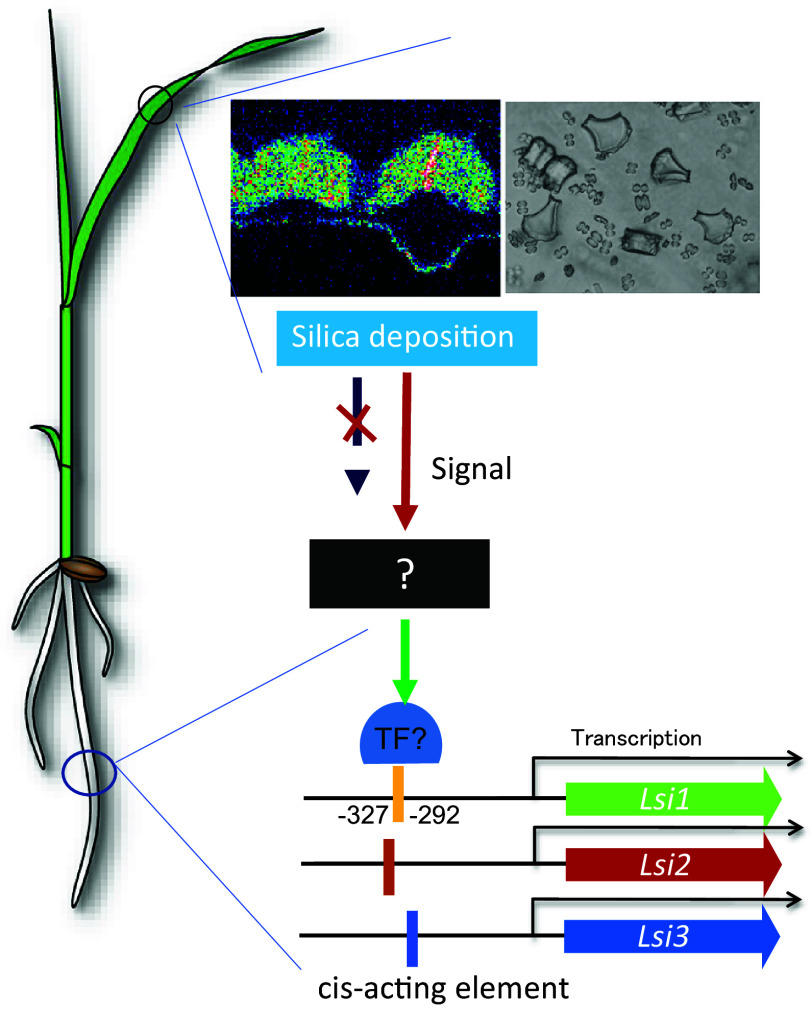
Proposed mechanisms for silicon-induced transcriptional regulation of *Lsi1, Lsi2* and *Lsi3* in rice. Silicon deposition in the leaf generates a signal, which is transported to the roots to suppress the expression of *Lsi1/2/3* through unknown pathway. Alternatively, a signal molecule generated in the leaves was suppressed due to silicon deposition, resulting the shutdown of the signal transport to the roots required for activating the expression of *Lsi1/2/3*.

A model for Si distribution in node I was also constructed (Yamaji et al., 2015). This model revealed that in addition to three transporters localized at the different cell layers in node I as described above, an apoplastic barrier at the bundle sheath, enhanced xylem area of enlarged vascular bundle, and folded plasma membrane of xylem transfer cells also contribute to the preferential distribution of Si to the grains, although at different extents (Figure 2B).

## 4. Si “homeostasis”

Rice is able to grow under both upland and flooded conditions, which differ greatly in Si concentration in soil solution. Moreover, in a single growing season, rice plants are usually exposed to regimes of flooding and drought several times for optimal productivity. Therefore, rice has to cope with fluctuations of Si in environments to maintain its “homeostasis”. In the case of most mineral elements, homeostasis is maintained by regulating transporters at the transcriptional or/and posttranscriptional levels (Ma & Tsay, 2021). However, different from other mineral elements, in which homeostasis is sensed by a change in cytosolic concentration, Si is mainly deposited in the apoplastic space as SiO_2_. Therefore, the so-called “homeostasis” of Si is different from other mineral elements. Among Si transporters identified so far, the expression of *Lsi1*, *Lsi2*, *Lsi3* and *Lsi6* was induced by Si deficiency (Ma et al., 2006, Ma et al., 2007; Huang et al., 2022), but that of *SIET4* was constitutively expressed (Mitani-Ueno et al., 2023). Studies have shown that no rapid degradation systems are involved in Si uptake mediated by Lsi1 and Lsi2 (Konishi et al., 2022), indicating that Si uptake is mainly regulated at the transcriptional level. Interestingly, this Si-induced down-regulation of *Lsi1*, *Lsi2* and *Lsi3* results from Si accumulation in the shoot rather than in the roots based on a root-split experiment (Mitani-Ueno et al., 2016). There was a good negative correlation between shoot Si accumulation and the expression level of *Lsi1* and *Lsi2*. These findings suggest that there may be two signal pathways for the transcriptional regulation of *Lsi1*, *Lsi2* and *Lsi3* (Figure 3). One is that Si deposition in the leaf generates a signal, which is transported to the roots to suppress the expression of *Lsi1/2/3* through an unknown pathway. The other one is that a signal molecule generated in the leaves was suppressed due to Si deposition, resulting in the shutdown of the signal transport to the roots for the expression of *Lsi1/2/3*. The promoter region of *Lsi1* between −327 to −292 was found to be responsible for its down-regulation (Mitani-Ueno et al., 2016), but the exact mechanism is not clear on how rice plants optimize Si accumulation (“homeostasis”) through regulating the expression of *Lsi1*, *Lsi2* and *Lsi3* (Figure 3).

## 5. Future perspective

Although great progress has been made in the identification of Si transporters during the last two decades, the whole picture of Si accumulation and regulation of Si transporters is still obscure. In terms of Si transporters, there may be some unidentified ones. For example, it is unknown how Si is released from the parenchyma cells following unloading by Lsi6 from the xylem in the leaves. It is also not clear how Si is further transported to specific tissues of leaves in addition to SIET4. Transporters responsible for the local distribution of Si between different tissues (e.g. leaf sheath versus leaf blade) have not been identified. The mechanisms underlying polymerization remain to be investigated in addition to the chemical polymerization of monosilicic acid at high concentrations. Some proteins such as PRP1 and Slp1 have been implicated in scaffolding silica in cucumber (Kauss et al., 2003) and sorghum (Kumar et al., 2020), respectively, but it is unclear whether a similar mechanism takes place in rice.

Since the discovery of Lsi1 and Lsi2 in rice, several homologs have been identified and functionally characterized in other several plant species differing in Si accumulation. However, Si transporters in most plant species have not been identified. Functional characterization of Si transporters like Lsi1 and Lsi2 in rice will help better understand the uptake system of Si in different plant species.

The feature of Si transporters (Lsi1 and Lsi2) in rice is their polar localization, which is required for efficient Si uptake in rice. Furthermore, the polar localization is cell-specific. One example is when *Lsi3* is expressed under the control of the *Lsi2* promoter, making non-polar Lsi3 have a polar localization (Huang et al., 2022). However, the exact molecular mechanism underlying the polar localization is poorly understood. It seems that the mechanisms of polar localization of Si transporters differ from those found for other transporters in Arabidopsis. For example, clathrin-mediated endocytosis is not required for the polar localization of Si transporters (Konishi et al., 2022).

Finally, it is clear that Si deposited in the shoots regulates *Lsi1*, *Lsi2* and *Lsi3* at the transcriptional level. However, many players involved in these processes need to be identified. For example, it is not clear how plants sense Si accumulation, what kind of signal is generated, and how this signal is transported and finally regulates the expression of *Lsi1/2/3*. Elucidation of these questions will help to understand how rice accumulates optimal Si (“homeostasis”) through the regulation of Lsi1, Lsi2 and Lsi3, key transporters for Si uptake and xylem loading in rice.

## Data and coding availability statement

There are no data to share.
