# Supplementary material for: Silicon transport and its “homeostasis” in rice
Source: Quant Plant Biol. 2025 Jan 9;5:e15. doi: 10.1017/qpb.2024.19 (PMC11729502; doi:10.1017/qpb.2024.19)
Supplement: Peer review history

# Editor report: Recommendation: Silicon transport and its “homeostasis” in rice — R0/PR2

**DOI:** 10.1017/qpb.2024.19.pr2

**Affiliations:** CBSM - Faculty of Engineering, Universidad de Talca, Chile

Dear Dr. Ma,

manuscript “Silicon transport and its ‘homeostasis’ in rice” was reviewed by two independent reviewers. Both are very positive in their assessment. One reviewer suggests a few “cosmetic” adjustments, which I ask you to consider in a minor revision.

Thank You for your valuable contribution to the the Research Topic “Quantitative approaches to cellular aspects of plant ion homeostasis”.

Best regards

Ingo Dreyer

# Editor report: Decision: Silicon transport and its “homeostasis” in rice — R0/PR3

**DOI:** 10.1017/qpb.2024.19.pr3

**Affiliations:** RDP, ENS de Lyon, France

No accompanying comment.

# Editor report: Recommendation: Silicon transport and its “homeostasis” in rice — R1/PR5

**DOI:** 10.1017/qpb.2024.19.pr5

**Affiliations:** CBSM - Faculty of Engineering, Universidad de Talca, Chile

Dear Jian Feng and Sheng Huang,

thank you for the careful revision of the manuscript. I agree with the reviewers stating “this information will be of great value”, “a pleasure to read”. Thanks again for your contribution. It is highly appreciated.

Best regards, Ingo

# Editor report: Decision: Silicon transport and its “homeostasis” in rice — R1/PR6

**DOI:** 10.1017/qpb.2024.19.pr6

**Affiliations:** RDP, ENS de Lyon, France

No accompanying comment.
